# Nurses at the helm of transforming lives in the shadows of Hemophilia

**DOI:** 10.6026/973206300212639

**Published:** 2025-08-31

**Authors:** Gnana Sarjana Ramakrishnan, Shankar Shanmugam Ragendran, Gomathy Priya Venkatachalam, Jebamalar Rajan, Gnana Malar Periyakaruppan, Herolin Vimala Muthupillai, Bibilin Wilson

**Affiliations:** 1 Department of Community Health Nursing, College of Nursing, Madras Medical College, The Tamil Nadu Dr. MGR Medical University, Chennai, Tamil Nadu, India; 2 Department of Child Health Nursing, College of Nursing, Madras Medical College, The Tamil Nadu Dr. MGR Medical University, Chennai, Tamil Nadu, India; 3 Department of Medical Surgical Nursing, College of Nursing, Madras Medical College, The Tamil Nadu Dr. MGR Medical University, Chennai, Tamil Nadu, India

**Keywords:** Hemophilia, quality of life, nurse-led intervention, chronic illness, mixed-methods study

## Abstract

The multifaceted challenges faced by hemophilia survivors, focusing on psychological distress, social isolation and quality of life (QoL). The intervention, a nurse-led program, significantly improved QoL by addressing emergency management, physical activity and dietary guidance. The quantitative results showed a notable reduction in QoL scores for the intervention group. Sociodemographic factors like age, disease severity and place of residence influenced outcomes. Thus, we show the importance of comprehensive, patient-centered care to improve both clinical and psychosocial outcomes for hemophilia survivors.

## Background:

Haemophilia is an X-linked recessive coagulation disorders characterised by deficits in clotting factors VIII (Haemophilia A) or IX (Haemophilia B), which hinders the body's capacity to form blood clots and results in extended bleeding episodes. This illness primarily affects males and appears in varied severities depending on the degree of clotting factor present [1]. Worldwide, around 1.125 million guys are impacted, with approximately 418,000 suffering from severe haemophilia, which considerably diminishes their quality of life [2]. In the United States alone, around 33,000 males are living with this illness [3]. Severe haemophilia frequently leads to spontaneous bleeding episodes, particularly in joints, resulting in haemophilic arthropathy-a degenerative joint disorder characterised by chronic discomfort and limited mobility [4]. These problems significantly impair physical functioning and add to disability. Additionally, the financial strain is enormous. A scoping review indicated that the annual expense for managing severe haemophilia may vary from $1,566 to over $700,000, contingent upon the treatment plan and geographical location [5]. Contemporary treatments like gene therapy exhibit potential, although accessibility is constrained and costly. The extensive ramifications of haemophilia encompass psychosocial difficulties. A comprehensive assessment in Central Europe revealed that 41.8% of patients reported mobility restrictions, 38.5% experienced unforeseen bleeding incidents and 36.6% endured chronic pain [6]. Mental health constitutes a significant concern. The CHESS II trial revealed heightened anxiety and depression levels in individuals with haemophilia A relative to the general population, underscoring the necessity for extensive psychological assistance [7]. Patients often articulate unhappiness with their healthcare owing to perceived deficiencies in provider expertise, which can intensify emotional suffering and hinder treatment adherence [8]. The most devastating long-term effect of haemophilia is joint arthritis. As many as 70% of patients limit physical activity due to discomfort and joint deterioration, resulting in diminished life satisfaction [9]. Similarly, research indicates that approximately 60% of individuals with haemophilia experience chronic discomfort, hence reducing their quality of life [10]. Nurse-led treatments have emerged as effective models for enhancing outcomes. These interventions generally encompass physical rehabilitation, patient education and emotional support. Nurse-developed exercise regimens have demonstrated efficacy in improving mobility and alleviating joint discomfort [11]. Nurse-led educational sessions markedly enhance self-efficacy and enable patients to proactively manage bleeding episodes [12]. Therefore, it is of interest to explore the health challenges and the impact of Nurse-Led Interventions on Quality of Life in Hemophilia Survivors.

## Materials and Methods:

This study employed a mixed-methods exploratory sequential design to comprehensively understand the health challenges faced by hemophilia survivors and evaluate the effectiveness of nurse-led interventions on their quality of life. Initially, qualitative data were gathered using a phenomenological approach to explore lived experiences, followed by a quasi-experimental, non-randomized control group design in the quantitative phase to measure intervention outcomes. The sample size for the quantitative study was derived at 58% of adolescents with hemophilia had low knowledge about their condition. Using a confidence level of 95% and a relative precision of 22%, the calculated sample size was 60 participants. This calculation applied the formula:

N=Z^2^ x (1-p)/p x e^2^

Where Z = 1.96, p = 0.58 and e = 0.22.

The study was conducted in the Hematology OPD of Rajiv Gandhi Government General Hospital (RGGGH), Chennai. The qualitative phase involved ten participants selected via purposive sampling. In-depth interviews were audio-recorded, transcribed and analyzed thematically using NVIVO software. The quantitative phase enrolled 60 participants through convenience sampling, divided equally into experimental (n=30) and control (n=30) groups. Pre-tests were conducted using a sociodemographic questionnaire, clinical data sheet and the Haemophilia Adult Quality of Life (Haem-A-QoL) scale. After 21 days, post-tests were administered to evaluate changes. The intervention consisted of a 40-45-minute structured session focusing on four key areas: emergency management, exercise, diet and daily activity modification. Participants were educated on recognizing and managing bleeding episodes, initiating timely care and understanding when to seek medical help. They received guidance on tailored exercises to improve joint mobility and reduce complications, along with nutritional counseling to support overall health. Practical strategies were also provided to safely perform daily tasks while minimizing injury risk. This holistic, patient-centered approach aimed to enhance self-management and improve quality of life. The control group received only routine hospital education. Data were entered into Microsoft Excel and analyzed using SPSS Version 22. Descriptive statistics summarized sociodemographic and clinical characteristics. Inferential analysis included paired t-tests for within-group comparisons and chi-square tests to examine relationships between quality-of-life scores and demographic variables. Thematic content analysis was used for qualitative data, ensuring data saturation and triangulation for validity. Statistical significance was set at p < 0.05.

## Results:

The mean age of the participants is 37.2 years with a SD of 11.24 years. Participants were predominantly male (100%) and aged between 18-60 years. The most common type of hemophilia was type A (experimental: 80%, control: 86.67%) and a majority were diagnosed before age five. The severity was mostly moderate, with joint problems reported as the most prevalent comorbidity. All participants had regular access to factor replacement therapy. No significant demographic or clinical differences existed between groups at baseline (p > 0.05), ensuring comparability. Thematic analysis of qualitative data from hemophilia survivors revealed nine major themes with accompanying subthemes that illustrate the multifaceted challenges of living with the condition. Emotional and Psychological Distress encompassed depression and helplessness, anxiety about future bleeds, emotional exhaustion from hiding pain, psychological trauma and lack of mental health support. One participant shared, "Some days I just lie in bed crying, feeling useless and tired of this pain" (Participant 3), while another noted, "They fix my bleed but never ask how I'm coping mentally" (Participant 4). Fear and Uncertainty around Parenthood and Genetic Transmission included anxiety about passing hemophilia to children, emotional impact of seeing children at risk and lack of access to genetic counselling. "I don't want my child to go through what I did" (Participant 1) reflected the generational burden. Identity Crisis Following Career Disruption highlighted loss of career, transition to dependency and emotional consequences of unemployment. "I worked so hard to become an engineer-now I just sit at home" (Participant 5). Barriers in Healthcare Access and Quality included lack of provider knowledge, delayed treatment and absence of integrated care, as captured by "They gave me wrong injections-it worsened the bleeding" (Participant 4).

Social Isolation and Stigma addressed withdrawal from social life, blood-related stigma and workplace discrimination. "People stare or ask strange questions, so I just stay home" (Participant 1). Impact on Family Dynamics and Relationships revealed guilt, parenting strain, emotional burden on caregivers and financial stress, with "We sold our land twice just to buy factor vials" (Participant 2). Genetic Anxiety and Family Planning and Loss of Independence and Role Identity-including role reversal, masculinity loss and feeling burdensome completed the themes. "Everyone adjusts for me-I feel guilty even asking for water" (Participant 5) encapsulated these struggles. At baseline, no participants in either group reported a high level of quality of life (QoL). The experimental group had 56.67% with moderate QoL and 43.33% with low QoL, while the control group had an equal 50% in both categories. Post-intervention, 66.67% of the experimental group achieved a high QoL and 33.33% had moderate QoL. The control group showed no high QoL scores, with 60% remaining at moderate and 40% at low QoL levels. These differences were statistically significant (χ^2^, p ≤ 0.001). The total QoL score improved significantly in the experimental group, from a mean of 179.50 to 120.23 (p < 0.001), while the control group saw a minimal, non-significant change (179.07 to 175.67). Domain-wise analysis showed highly significant improvements in all areas, including physical health, emotional well-being and treatment satisfaction (p < 0.001) (Table 1 - see PDF).

## Discussion:

This study explored the lived experiences of hemophilia survivors, assessed their baseline quality of life (QoL), evaluated the impact of nurse-led interventions and examined associations with demographic variables. The study identified nine major themes that encapsulate the lived experiences of hemophilia survivors. These include Emotional and Psychological Distress, Fear and Uncertainty Around Parenthood and Genetic Transmission, Identity Crisis Following Career Disruption, Barriers in Healthcare Access and Quality, Social Isolation and Stigma, Impact on Family Dynamics and Relationships, Genetic Anxiety and Family Planning, Loss of Independence and Role Identity and a reiterated theme of Social Isolation and Stigma, underscoring its pervasive impact across personal and social domains. These findings are consistent with those of Ramos-Petersen *et al.* who reported daily struggles, uncertainty and poor institutional support among hemophilia patients [13]. Similarly, Arya *et al.* emphasized identity disruption, evolving treatment practices and barriers to care as key themes in Indian men with hemophilia [14]. Pre-intervention QoL assessments showed no participants had a high level of QoL, aligning with studies in China and India reporting that most hemophilia patients experience moderate to poor QoL, despite treatment availability [15]. These results highlight the limitations of pharmacological therapy alone in addressing psychosocial and functional aspects of the disease. Following the nurse-led intervention, the experimental group demonstrated a 33.77% improvement in QoL, compared to only 1.48% in the control group. These findings strongly support the effectiveness of structured, personalized nursing programs. Torres *et al.* similarly observed significant QoL improvements in patients with bleeding disorders following nurse-led education and psychosocial support [16]. López-Casaus *et al.* further validated this approach, reporting enhanced health outcomes in hemophilia patients through pain management, exercise guidance and emotional counseling delivered by nurses [17]. Post-test associations revealed that place of residence, age at diagnosis and disease severity significantly influenced QoL outcomes, favouring urban residents, early diagnosis and moderate disease severity. These findings are in line with those of Schmidt *et al.* who identified age, disease severity and physical activity as determinants of QoL among hemophilia patients [18]. Likewise, Moka *et al.* found significant associations between diagnosis duration, geographic residence and health-related QoL [19]. This study confirms that hemophilia imposes complex emotional, social and systemic challenges. Nurse-led interventions significantly enhance QoL and sociodemographic factors influence these outcomes when structured care is provided.

## Conclusion:

Hemophilia survivors experience complex challenges impacting emotional, social and physical well-being. Nurse-led interventions effectively enhance quality of life by addressing both clinical and psychosocial aspects. Thus, we show the importance of personalized care in improving outcomes and empowering long-term management.
